# Looking Beyond Pure Cellulose to Lignocellulose for Regenerated Continuous Spun Filaments

**DOI:** 10.1021/acsomega.5c10782

**Published:** 2025-12-13

**Authors:** Chinomso M. Ewulonu, Stefania Akromah, Koon-Yang Lee, Annela M. Seddon, Cariny Polesca, Jason P. Hallett, Stephen J. Eichhorn

**Affiliations:** † Bristol Composite Institute, School of Civil, Aerospace, and Design Engineering, 1980 University of Bristol, Bristol BS8 1TR, United Kingdom; ‡ Department of Aeronautics, 4615 Imperial College London, South Kensington Campus, London SW7 2AZ, United Kingdom; § H.H. Wills Physics Laboratory, School of Physics, University of Bristol, Bristol BS8 1TL, United Kingdom; ∥ Department of Chemical Engineering, Imperial College London, South Kensington Campus, London SW7 2AZ, United Kingdom; ⊥ School of Chemistry, Faculty of Science and Engineering, Cantock’s Close, University of Bristol, Bristol BS8 1TS, United Kingdom

## Abstract

The need to use naturally abundant, renewable, and sustainable precursors, such as lignin and cellulose, to produce technical textile fibers for a range of applications is rapidly growing. Being able to spin fibers directly from the biomass feedstock, without separation and purification, could significantly reduce processing costs, energy consumption, and pollution, and also retain carbon for subsequent use in carbon fiber production and other applications. Going beyond the approach of either spinning pure lignin, cellulose, or combinations of the two, continuous regenerated spun fibers have been successfully produced from dissolved and unbleached miscanthus grass pulp. The rheological and microscopic properties of the spinning dope were fully characterized as well as the structure and mechanical properties of the spun lignocellulose pulp (LCP) fibers. The highly viscous spinning dope had a zero-shear viscosity in the range 26–256 kPa·s, which resulted in spun fibers with a rough surface texture, with some undissolved lignocellulose components in the dope. The LCP fiber’s orientation was determined using X-ray diffraction, displaying low- to mid-range values of <sin^2^ θ> (0.2–0.5), which was expected at the low draw ratios used to ensure fiber consistency. Despite this, the filaments were found to have strengths in the range of 114–173 MPa, similar to wool or wet viscose rayon, and moduli of 9–12 GPa comparable to lower-range lyocell fibers. Interestingly, the micrometer-scale undissolved lignocellulose components did not inhibit the spinning process, allowing the production of what resembles continuous natural fibers. This approach shows promise for generating sustainable continuous spun fibers, without excessive pretreatment of the precursor, for technical textiles from lignocellulose pulps.

## Graphical abstract

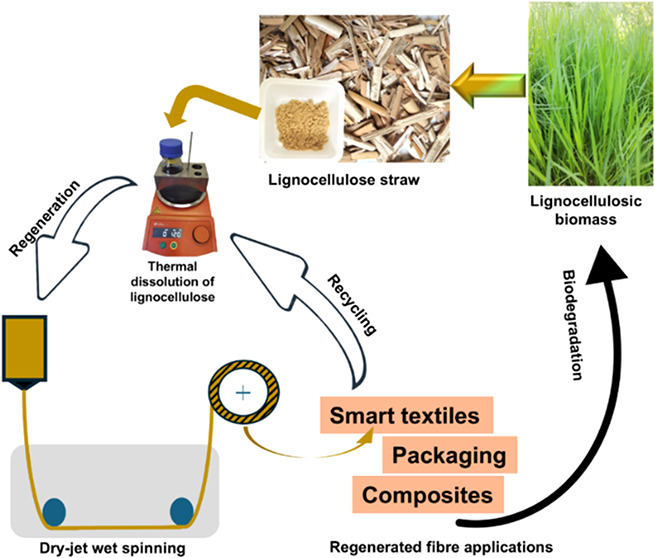

## Introduction

Regenerated cellulose fibers are used in everyday human life from clothing, packaging, storage, building materials to composites for automotive applications.^1–3^ Historically regenerated cellulose fibers have been used as a precursor for carbon fibers,^4^ even subsequently finding their way into some aerospace applications.^5^ Cellulose, being abundantly available in nature, is considered as a sustainable feedstock to replace petroleum and fossil-fuel-based feedstocks^2,6,7^ in fiber production. Cellulose has been prepared in several forms for the production of regenerated cellulose fibers, e.g., microcrystalline cellulose,^8,9^ cellulose nanofibrils,^10^ and bleached pulps.^11,12^ The spun fibers are produced by first dissolving the cellulose in a solvent, followed by spinning, typically using a dry-jet wet process. Although this seems a straightforward process, cellulose processing is challenging because it does not melt, and it is insoluble in many conventional solvents.^13^ The insolubility of cellulose is thought^14^ to arise from the extensive inter- and/or intramolecular hydrogen bonding in its structure, but also other interactions (van der Waals’ bonds, hydrophobic interactions) are known to play a role too.^15^

Presently, the two widely adopted industrial technologies (viscose and lyocell) for generating regenerated cellulosic fibers have issues relating to the toxicity and safety of the chemicals employed in dissolution. These issues include the formation of gases harmful to workers (viscose production) and the environment^16^ and the poor thermal stability of the solvent (lyocell production).^16^ The degradation of both the solvent and the cellulose due to the formation of byproducts, high safety costs, complex dissolving techniques, tough solvent recovery procedures, and low productivity due to the low solubility of cellulose in the (highly toxic) *N*-methyl morpholine-N-oxide (NMMO monohydrate)^17,18^ solvent has led to the search for ‘greener’ approaches for efficient cellulose dissolution. Cellulose also normally has to be derivatized first, before dissolution can take place, e.g., the production of soda-cellulose for the viscose process. Ionic liquids (ILs) have emerged as revolutionary and potentially green solvent systems for the dissolution of cellulose. They also allow dissolution from nonderivatized material. ILs, particularly those containing imidazolium, pyridinium, or ammonium-based cations and halide or acetate anions, can disrupt the extensive hydrogen bonds in cellulose enabling its dissolution under mild conditions.^19–21^ Interestingly, the use of ILs avoids harsh chemicals and minimizes environmental impact, as ILs have low volatility and are easily recoverable after regeneration of the cellulose material.^22,23^ It has further been demonstrated^24^ that 1-ethyl-3-methylimidazolium diethyl phosphate [EMIm]­[DEP] can be recovered and reused 5 times to pretreat wheat straw, with over 50% yield. Another IL, 1-ethyl-3-methylimidazolium acetate [EMIm]­[AC], has demonstrated a 90% conversion rate of miscanthus cellulose to glucose after a second recycle.^25^ Notably, the use of ILs can require little or no pretreatment of lignocellulosic biomass, simplifying the process and reducing energy consumption.^19^ However, not all ILs can dissolve cellulose and their dissolution efficiency can vary considerably^13,20^ and concerns about the potential toxicity of ILs are also limiting their large-scale adoption.^26^ Cellulose-based fiber spinning processes using ILs have been previously critically reviewed.^16,27^

The conventional methods of obtaining a suitable refined cellulose material for fiber spinning have many environmental issues of their own. These methods involve the use of various physical or chemical processes to remove the lignin and hemicellulose, which are often seen as a problem when it comes to making continuous fibers. These processes have been described to be cost-ineffective,^28^ energy-consuming, and detrimental to the environment.^29^ The numerous challenges associated with the pretreatment of lignocellulose biomass have been previously reviewed.^30^ Many other research groups,^31–33^ while weighing these gains and shortfalls, believe that lignin—which is the major component removed from these processes—can positively contribute to the improvement of the properties of cellulose and its applications. It is important to note that many applications that utilize pure cellulose do not need it in its pure state for processing. These applications include, for example, technical textile fibers incorporated in composite materials for automotive parts, construction materials, and packaging or adsorbent materials for removing heavy metals and dyes from wastewater. In more recent times, much research has focused on the use of lignin (which has higher carbon content) to cellulose to produce carbon fibers (CFs).^8^ The challenge with this approach lies in the high energy, chemical, water, and technical expertise required to separate lignin from the cellulosic biomass at the source. This then requires additional chemicals and processing to recombine them into a viable precursor, which adds cost to the whole process.

The use of wood pulps (dissolving pulps) for the production of a suitable precursor material to make regenerated cellulose fibers necessitates high costs. This is because of the significant time and resources required to cultivate the forests, which do not typically operate on an annual growth basis. Miscanthus is a perennial grass known for its rapid growth and high biomass yield,^34,35^ making it ideal for sustainable fiber production. Agronomically, miscanthus requires lower inputs for field operations, low maintenance cost, and regeneration compared to other crops, such as those needed for traditional wood pulp production.^36^ Its ability to thrive on marginal lands, which are often unsuitable for food crops, highlights its potential for cultivation with minimal economic impact on existing agricultural markets.^36,37^ This flexibility not only helps in preserving arable land for food production but also promotes biodiversity by reducing the dependence on monoculture farming systems.^36,38^ Additionally, miscanthus reaches maturity quickly, can be reharvested annually,^37^ and as a C4 plant (a plant that uses a specialized photosynthetic pathway, known as C4 photosynthesis, to maximize photosynthesis and minimize water loss, making them well suited for challenging environments),^39,40^ miscanthus is highly efficient in sequestering carbon dioxide, thereby contributing to the reduction of greenhouse gas emissions associated with fiber production processes.^41,42^ This aligns with global efforts to mitigate climate change impacts by reducing the carbon footprint of industrial activities.

In this work, miscanthus grass is used as the lignocellulose biomass source, as we hypothesize that regenerated spun lignocellulose (LC) filaments can provide equivalent mechanical and thermal properties required of cellulose filaments in similar technical textile applications. The miscanthus grass (partially treated with NaOH) is dissolved in 1-ethyl-3-methylimidazolium diethyl phosphate ([EMIm]­[DEP]) IL and regenerated as a single continuous filament through the dry-jet wet spinning process, yielding fibers with comparable properties to those currently on the market. The use of [EMIm]­[DEP] is based on previous reports^9,10,43,44^ on its efficacy in dissolving different cellulose types. We do not aim to produce high-performance fibers using this approach, only demonstrating that it is possible to make filaments with properties in the range of commercial forms, but critically using the whole lignocellulose feedstock.

## Experimental Section

### Materials

Miscanthus pulps, partially treated with sodium hydroxide, were provided by ESG Natural Capital, Taunton, U.K. The properties of the pulp are reported in subsequent sections. The lignocellulose pulps (LCP) were processed using a conventional kitchen blender, and oven-dried at 60 °C until a consistent moisture content between 1 and 2% was achieved. 1-Ethyl-3-methylimidazolium diethyl phosphate ([EMIm]­[DEP]) with purity >98% from Iolitec, GmbH was used as purchased. The commercial cellulose pulp (CCP) used was in the form of dried cellulose sheets, derived from eucalyptus wood supplied by Fiberlean Ltd. (Par, U.K.). The compositional analysis of the pulps and fiber filaments was carried out according to the standard National Renewable Energy Laboratory (NREL) analytical procedure.

### Spinning Dope Preparation

The spinning dopes were prepared by dissolving the LCP in [EMIm]­[DEP] at 120 °C for 20 h. Briefly, 2.5, 5, 7.5, and 10 g of LCP with moisture contents less than 2% were added to 50 g of previously heated [EMIm]­[DEP]. Moisture was kept to a minimum as this acts as an antisolvent for most ILs. For this reason, the glass reaction vessel was sealed to prevent moisture from interfering with the dissolution and stirred intermittently. The resulting spinning dopes were labeled LCP5, LCP10, LCP15, and LCP20, respectively, to illustrate the percentage of lignocellulose pulp in the IL. CCP in sheet form was also processed with a kitchen blender and dissolved using the same procedure. Only 5 g (10%) of CCP was dissolved in the IL, and heated at 120 °C for 4 h (full dissolution was observed within this time) to compare the properties obtained to the LCP series of fibers.

### Characterization of Spinning Dope

A small amount of the spinning dopes was deposited on a glass slide and observed under an optical microscope (Zeiss AX10 Imager.M2, U.K.). The viscosity of the spinning dope was measured using a Discovery HR-1 rheometer (TA Instruments) following a previously reported method.^8^ Logarithmic sweep steady shear rheometric studies were carried out at 30, 60, 80, 100, and 120 °C using shear rates in the range 0.001–100 s^–1^ to determine the dopes’ effective spinning temperatures. Oscillatory tests were performed at 5% strain at 80 °C within an angular frequency range of 0.01–100 rad s^–1^ to determine the viscoelastic properties of the LC spinning dope.

### Dry-Jet Wet Spinning

Monofilaments were spun from the LC spinning dope using bespoke equipment that has been previously described.^8–10^ Spinning was carried out at a constant temperature of 80 °C. The airgap was maintained at 20 mm, and the LC monofilaments were extruded through a nozzle with an internal diameter of 413 μm. Initially, the LC spinning dope was conditioned at 80 °C in the extruder barrel for 1 h to ensure an even equilibration of the dope temperature before extruding at a linear velocity of 0.62 m s^–1^, while the winder linear velocity was set at 0.31 m s^–1^, resulting in a low draw ratio of 0.5 to prevent fiber breakage, which would occasionally occur at higher draw ratios. This ensures that essential baseline data for the spinning of LC dopes are obtained since dope modification may be required for a higher draw ratio to be used. Excess IL, after fiber spinning, was removed from the spun LC filaments by immersing them in water at room temperature for 24 h with a change of water twice within this period. The complete removal of the IL was not possible for the LCP fibers, and Fourier transform infrared spectroscopy (FTIR) analysis showed the presence of [EMIm]­[DEP] residual bands (∼1575 cm^–1^) for these samples. The LC filaments were air-dried, remaining wound on the roller to exert a little tension and prevent their shrinkage.

### Characterizations of the LC Spun Filaments

The surface topography, cross-sectional morphology, and diameters of the LC spun filaments were investigated using a JSM IT300 JEOL (Japan) scanning electron microscope. Images were collected for each sample, and diameter measurements were taken from 20 different locations on each filament using ImageJ software. Statistical analysis of these data was undertaken using Minitab Statistical Software 22.

FTIR analysis was used to investigate the chemical compositions of the fibers. The spectra obtained from the fibers were compared with those of EMImDEP and LCP to confirm the removal of the IL after spinning. Each spectrum was collected using 64 scans at a 4 cm^–1^ resolution between 4000 and 600 cm^–1^ in absorbance mode with an air background considered in the analysis. Wide-angle X-ray diffraction (WAXD) measurements of the LC spun filaments were performed^9^ using a SAXSLAB GANESHA 300 XL instrument, with an exposure time of 600 s. Data were reduced by using SAXSGUI software.

### Mechanical Properties and Thermal Stability

Tensile modulus, strength, and elongation at break of the LC spun filaments were determined from mechanical property measurements made on a 1 kN Instron 3343 tensile tester in accordance with ASTM D3822-01. These properties were obtained from an average of 10 measurements for each sample using three gauge lengths (20, 40, and 60 mm). The strain rate for the 3-gauge lengths was maintained constant at 0.125 min^–1^ by altering the speed of deformation appropriately. The calculations of the means, standard deviations, and ANOVA statistical tests are presented in the Supporting Information. Initially, the linear density of the LC spun filaments was calculated in terms of tex, defined as the weight in grams per 1000 m of the LC spun filaments (see the Supporting Information for details). The tenacity of the LC spun filaments was calculated thus using the equation

1 $$tenacity\;\left(cN/tex\right)=\frac{breaking\;force\;\left(N\right)\times 100}{linear\;density\;\left(tex\right)}$$

The thermal stability of LC spun filaments was evaluated using a Netzsch STA 449 F3 (Germany) instrument with samples in a nitrogen environment. LC samples were cut into small pieces of about 10 mg and loaded into the STA crucibles, which were subsequently heated at a rate of 10 °C min^–1^ from 25 to 900 °C. Changes in mass as a function of temperature were automatically recorded.

## Results and Discussion

### Physical Properties of the Spinning Dope

Optical micrographs (OM) of the prepared dopes (Figure 1) show the undissolved fibers in various shapes and sizes. The OM of CCP, presented in Figure 1a, shows densely packed, long fibers. Due to the extensive industrial pretreatment of CCPs to remove lignin and hemicelluloses, they were completely dissolved in [EMIm]­[DEP] as seen in Figure 1b. On the other hand, the LCP, shown in Figure 1c, which did not undergo pretreatment, was found to incompletely dissolve. The undissolved particles (seen in Figure 1d) may be attributed to the presence of lignin and hemicellulose that may inhibit the dissolution of the cellulose fraction. Figure 1b confirms previous reports^8–10^ that [EMIm]­[DEP] is a good solvent for the dissolution of pure cellulose pulp.

**Figure 1:**
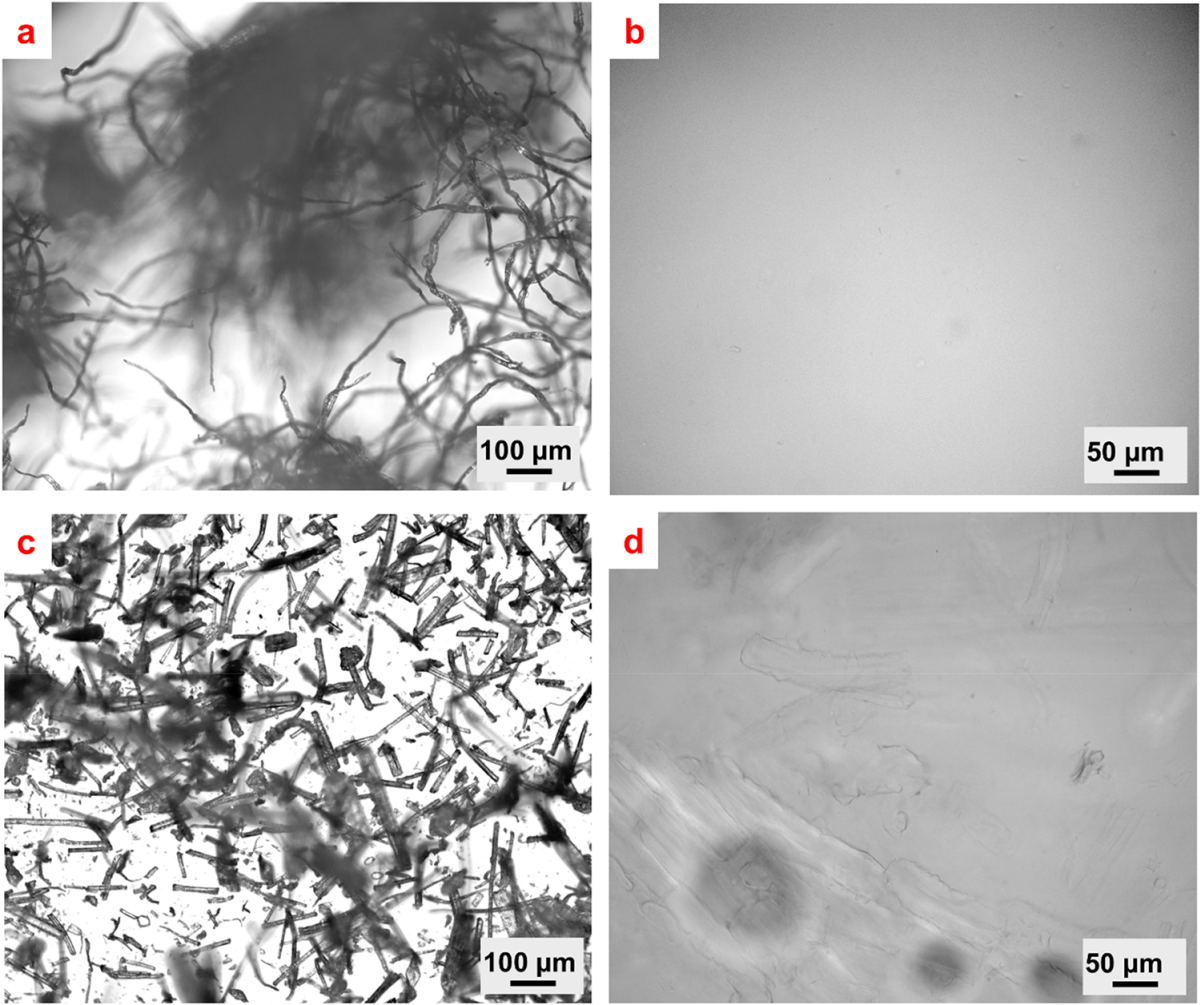
Typical optical micrographs of (a) CCP before dissolution, (b) CCP after dissolution, (c) LCP before dissolution, and (d) LCP5 after dissolution in the IL [EMIm]­[DEP].

However, this IL may be inadequate to completely dissolve all of the lignocellulose components in one pot under the investigated conditions. Notably, the aim of this work was to study how to use all the components of the biomass to effectively reduce the chemical processing needed for fiber production but to also retain carbon throughout.

### Spinning Dope Rheological Properties

The rheological measurements of the lignocellulose and commercial cellulose dopes presented in Figure 2a show that they all exhibit shear thinning behavior. Since serrated plates were used, we are assured that this behavior is due to the dope properties and not slip at the plates as has been described recently.^45,46^ Their zero-shear viscosities (η_0_) obtained from the flow-sweep plateau at 0.001 s^–1^ shear rate and 80 °C are presented in Figure 2b. Zero-shear viscosity (η_0_) represents the limiting viscosity at infinitesimally low shear rates, providing crucial information about polymer molecular weight and chain entanglement behavior.^47^

**Figure 2:**
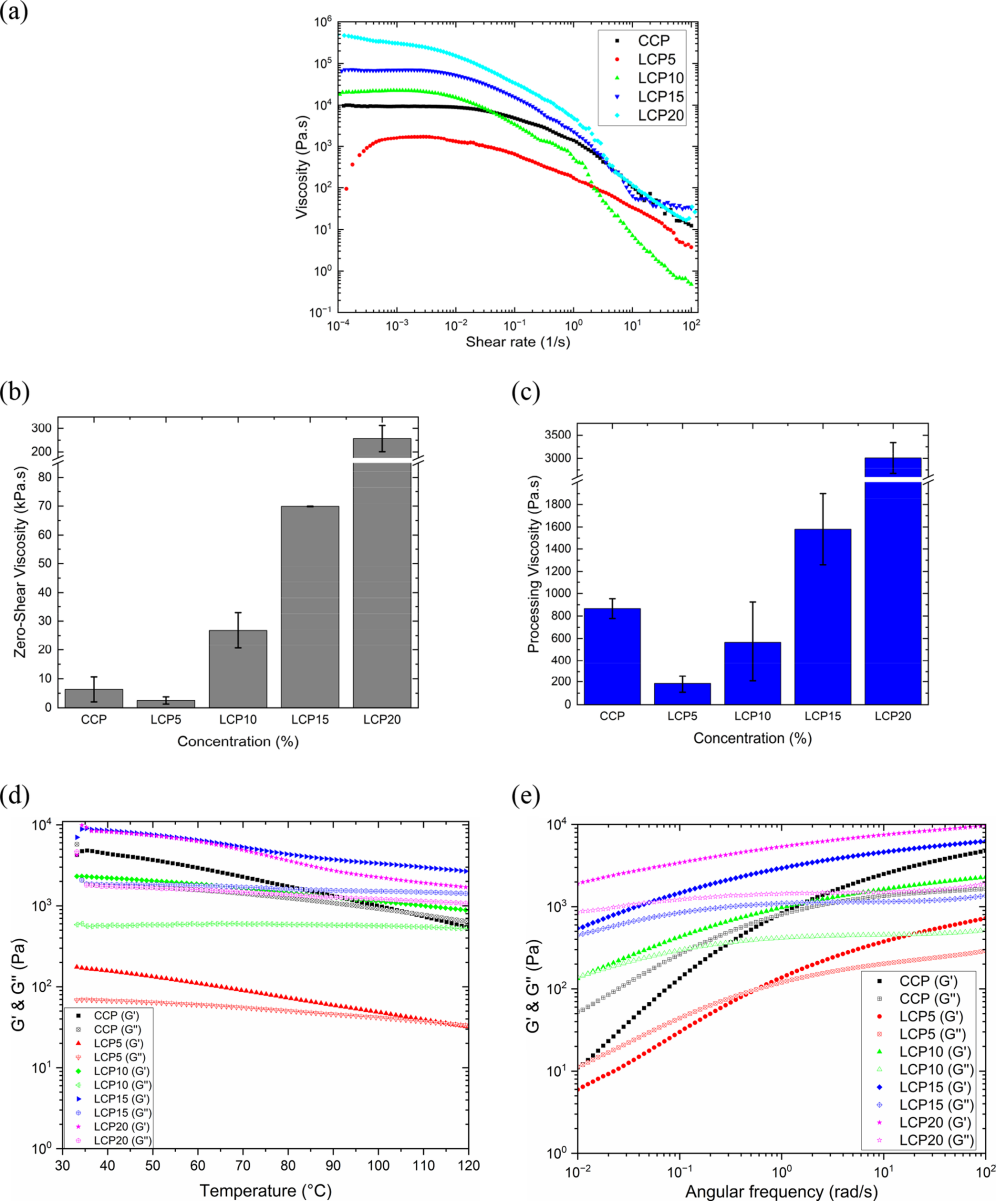
Rheological properties of the spinning dopes. (a) Change in viscosity with shear rate. (b) Zero-shear viscosity of spinning dopes at a processing shear rate of 1.6 s^–1^ and a temperature of 80 °C. (c) Processing viscosities of the spinning dopes. (d) Change in moduli (G' and G'') with angular frequency of the spinning dopes and (e) the change in moduli (G' and G'') with temperature of the spinning dopes.

Evidently, the zero-shear viscosities (Figure 2b) of the spinning dopes were higher with an increased concentration of LC, and all had a higher value than the CCP dope, except for the LCP5 dope. The LCP5 dope’s zero-shear viscosity at 80 °C was 2.5 ± 1.2 kPa·s, compared to the CCP dope with a viscosity of 6.3 ± 4.3 kPa·s. The zero-shear viscosities of LCP10, LCP15, and LCP20 are 25.9 ± 4.7 kPa·s, 69.9 ± 0.1 kPa·s, and 256.4 ± 55.2 kPa·s, respectively, which are an order of magnitude higher than the CCP dope. Obviously, these much higher viscosities of the LC pulp dopes will not only be attributed to their increased concentration, but also to the presence of undissolved fibers in the solution. Figure S1a–c (Supporting Information) demonstrates that LCP10, LCP15, and LCP20 contain relatively more undissolved fibers than LCP5 (Figure 1d), which could explain the difference in the viscosities. Although LCP5 does contain undissolved fibers, the quantity is thought to be not enough to outweigh the effect of the dissolved concentration, which is why it has a lower zero-shear viscosity than the CCP dope. The number-average and weight-average degrees of polymerization (DP) of untreated miscanthus have previously been reported^48^ to be 419 and 3987, respectively. This high DP confirms the viscosities we report, since viscosity is generally proportional to DP. While miscanthus ionoSolv pulps have also been noted^48^ to have a lower number-average DP and a competitive weight-average DP compared to commercial dissolving grade pulp, our interest lies in understanding the spinnability of untreated miscanthus.

Previous studies^49,50^ have suggested that the optimum spinning window for cotton waste cellulose dopes at 65–70 °C is within a range of 22–41 kPa·s of zero-shear viscosity. However, another study^51^ reported that 11.5% crushed fine paper dissolved in 1,5-diazabicyclo[4.3.0]­non-5-enium acetate IL with 16.5 kPa·s of zero-shear viscosity at 73 °C was spinnable outside this range. Only LCP10 falls within this range, and CCP and LCP5 fall just outside the lower end. Nevertheless, CCP could be spun by using the dry-jet wet spinning process presented here. LCP5 was not spinnable, with many breakages occurring. LCP15 and LCP20, which exhibited viscosities above the upper end of the range, were spun successfully into continuous filaments. More insights into the dope’s behavior while spinning can be understood from the relationship between viscosity and temperature at the processing shear rate, as illustrated in Figure 2c. The processing shear rate was obtained from the capillary shear rate equation. With a known extrusion speed of 5 mm min^–1^ and nozzle diameter of 413 μm, the processing shear rate (1.61 s^–1^) was calculated, and the respective viscosity was extracted from the flow-sweep curve using the equation

2 $$\gamma =\frac{4Q}{\pi R^{3}}$$

where *Q* is the volumetric flow rate (m^3^ s^–1^), *R* is the radius of the nozzle (m), and γ is the shear rate (s^–1^). *Q* is also expressed in terms of extrusion speed as

3 $$Q=A\upsilon =\pi R^{2}\upsilon$$

where *A* is the cross-sectional area of the nozzle, assuming a circular geometry (π*R* ^2^), and υ is the extrusion velocity (m s^–1^).

The viscosity at the processing shear rate (1.6 s^–1^) decreased significantly owing to the increased shear rate due to the shear thinning behavior of the dope. With an increase in concentration, there was an increase in viscosity within the range 184–3008 Pa·s. LCP5, which has the lowest processing viscosity (184 ± 76.3 Pa·s), was not able to produce stable continuous filaments without breakages. The low viscosity may contribute to the breakages, and subsequent low mechanical properties,^52^ but this is also linked to the lack of chain entanglement required for fiber production. These issues can be ameliorated by adjusting other parameters^53^ like concentration and temperature^50^ and through the use of spinning aids. Notably, we chose not to use spinning aids, e.g., poly­(vinyl alcohol), in the production of our fibers. Some reports have shown that the presence of native lignin in spinning dopes can restrict the motion of the polymer chains and enhance chain entanglements due to lignin-carbohydrate linkages.^54,55^ The LCP dopes, which were obtained from partially treated unbleached pulps, contain lignin, which may have also contributed to their high viscosities (Figure 2b).

An oscillatory temperature ramp test (Figure 2d) was performed on the spinning dopes to evaluate their thermal transitions and ensure their structural integrity under spinning conditions. When keeping strain constant at 5% and angular frequency at 1 rad s^–1^, heating the dopes from 30 to 120 °C revealed that their storage moduli decreased with a rise in temperature. The cross-over points (COP) for the CCP and LCP5 dopes were at 106 °C, 826 Pa and 116 °C, 34 Pa, respectively. This implies that the CCP dope has a lower softening temperature but a higher structural rigidity before breakdown compared to that of the LCP5 dope. This may result in lower mechanical properties for fibers obtained from the LCP5 dope and CCP dope degradation when processing at temperatures higher than 100 °C. Storage modulus (*G*′) was consistently higher than the loss modulus (*G*″) for the other spinning dopes. LCP10, LCP15, and LCP20 demonstrate structural stability at higher temperatures since *G*″ remains relatively constant while G′ gradually decreases with an increase in temperature but still remains higher than *G*″.

Moreover, Figure 2e demonstrates that the COPs of LCP20 and LCP15 occurred at very low angular frequencies (below 10^–2^ rad s^–1^), not covered in the experiment. CCP, LCP5, and LCP10s COPs occurred at 774 Pa at angular frequencies of 0.89 rad s^–1^, 98 Pa at 0.56 rad s^–1^, and 144 Pa at 0.01 rad s^–1^, respectively. This indicates that CCP undergoes increased chain entanglement, forming a stronger polymer network structure^56^ than the LCP dopes. This effect would itself result in fibers with increased mechanical properties, since chain entanglement is known to have a strong effect on these. The low modulus of the LCP5 dope can be explained by the low concentration of biomass polymers in the dope, making it relatively fluid-like with a weak network structure^50^ which may result in inadequate viscoelasticity required for spinning.^57^ The identification of COPs helps to understand viscoelastic properties, which relate to processability and the degree of physical or chemical cross-linking of spinning dopes.^58^ Remarkably, increasing the dope concentration leads to lower COP frequencies and higher moduli,^59^ which could result in increased mechanical performance of the fibers. However, this may also result in difficulties in spinning the filaments, which is why a compromise between the viscosity and performance is important.

### Chemical and Spun Filament Structural Features

The compositional analysis of the spun fibers and precursor biomass, including the CCP, is presented in Table 1. The presence of about 2% extractives and 4% mannose in the raw miscanthus fiber clearly differentiates it from the CCP, partially treated pulp, and spun fibers. Notably, up to 69% of the total lignin from the raw miscanthus fiber is present in the starting material (LCP). The LCP spun fibers had between 54 and 58% of the total lignin from the raw fiber after processing, this being a 17–22% decrease in the lignin content from the starting LCP pulp. This suggests that some material may be lost during the processing. In comparison, LCP had ∼15% more total lignin in its composition than the CCP material, and a higher hemicellulose content than CCP and the spun CCP fiber. The CCP fiber, which has ∼5% total lignin, only showed the presence of xylose, while the LCP spun fibers have both xylose and arabinose, with LCP also having only xylose. Regarding the lignin content, it is important to highlight that this procedure was optimized for raw biomass, and when using samples with low lignin content, the exposure to sulfuric acid can cause degradation of the sugars that are further identified as lignin in the analysis.^60^ The presence of lignin and especially hemicellulose in both starting materials (CCP and LCP) may have an effect on their level of dissolution. Spinning dopes from LCP with 50% hemicellulose more than the CCP showed incomplete dissolution, which obviously contributed to their performance.

**Table 1:** Compositional Analysis of the Spun Fibers and the Raw Fiber^a^; LCP Lignocellulose Pulp, CCP Commercial Cellulose Pulp, CCP Fiber, and LCP5-20 Spun Fibers from Commercial Cellulose and Lignocellulose Pulps

sample	extractives	^b^ASL (%)	^c^AIL (%)	total lignin (%)	ash (%)	glucose (%)	xylose (%)	arabinose (%)	mannose (%)
raw fiber	2.3 ± 0.0	7.8 ± 0.5	22.0 ± 1.2	29.8 ± 0.6	3.0 ± 0.4	40.7 ± 1.2	19.8 ± 0.6	0.0	4.4 ± 3.6
LCP	0.0	7.1 ± 0.4	13.5 ± 0.5	20.6 ± 0.3	0.4 ± 0.4	58.1 ± 0.6	20.9 ± 0.1	0.0	0.0
CCP	0.0	5.3 ± 0.3	0.0	5.3 ± 0.1	0.0	81.0 ± 0.2	13.7 ± 0.1	0.0	0.0
CCP fiber	0.0	5.7 ± 0.0	0.0	5.7 ± 0.0	0.0	77.0 ± 0.1	17.3 ± 0.2	0.0	0.0
LCP5	0.0	7.0 ± 0.1	10.4 ± 0.4	17.4 ± 0.2	0.4 ± 0.1	58.6 ± 0.4	19.8 ± 0.1	3.8 ± 0.1	0.0
LCP10	0.0	6.7 ± 0.2	9.6 ± 0.1	16.3 ± 0.1	0.0	57.8 ± 0.3	22.0 ± 0.2	3.9 ± 0.0	0.0
LCP15	0.0	6.4 ± 0.3	10.5 ± 0.0	16.9 ± 0.1	0.2 ± 0.2	56.4 ± 0.5	22.6 ± 0.0	3.9 ± 0.0	0.0
LCP20	0.0	6.9 ± 0.2	9.1 ± 0.2	16.0 ± 0.1	0.0	57.1 ± 0.4	23.0 ± 0.1	3.9 ± 0.1	0.0

a LCP, lignocellulose pulp; CCP, commercial cellulose pulp; CCP fiber, and LCP5-20 spun fibers from commercial cellulose and lignocellulose pulps.

b ASL, acid-soluble lignin.

c AIL, acid-insoluble lignin. All errors are standard deviations from the mean.

FTIR spectra of CCP, LCP, [EMIm]­[DEP], and the spun fibers are presented in Figure 3. Characteristic absorption bands differentiating the major chemical groups of lignin, hemicellulose, cellulose, and [EMIm]­[DEP] are identified. The sharp narrow band located at ∼897 cm^–1^ appeared for all the pulp and fiber samples, being absent only for [EMIm]­[DEP], as it is assigned to the β-glycosidic linkage vibrations of cellulose.^61^ [EMIm]­[DEP] is uniquely identified from the presence of a band located at ∼1240 cm^–1^, assigned to a phosphate P=O stretching mode.^62^ This band should not be confused with another present at ∼1224 cm^–1^, which is assigned to the aryl ether C–O stretching linkages of guaiacyl/syringyl units of lignin.^63,64^ This band was present only in the spectra for the LCP fibers and absent in the LCP pulp used to obtain the spun fiber. This difference is attributed to chemical treatments that had been applied prior to fiber spinning. These treatments degrade lignin’s β-O-4 aryl ether linkages, reducing their detectability in FTIR.^65^ However, the structural reorganization of lignin during regeneration^66^ or concentration effects due to its redistribution in the fiber matrix^67^ have evidently made the signal from the aryl ether C–O linkages more pronounced. Furthermore, the aromatic C=C skeletal vibrations in lignin are unambiguously detected only for the lignocellulosic pulps and its spun fibers, with a band located at ∼1506 cm^–1^. This band is characteristic of the presence of lignin in the pulp, which clearly distinguishes it from the CCP. The imidazolium ring of [EMIm]­[DEP] is prominently identified by the unique sharp band located at ∼1575 cm^–1^, assigned to C–N stretching. Notably, traces of [EMIm]­[DEP] detected at ∼1575 for the LCP fibers may have resulted from their surface morphology, allowing easy diffusion of the IL during washing or extended lignin-IL interactions. This further suggests that the LCP fibers may require extended washing periods since the band was not detected for the CCP fibers. Other unique bands are located at ∼1740 and ∼3150 cm^–1^, assigned to C=O stretching in acetyl or ester groups of hemicelluloses and aromatic C–H stretching of the imidazolium ring of [EMIm]­[DEP], respectively. Like the band for the aryl ether of lignin, the weak acetyl or ester band appeared only for the spun fibers, a further indication that the chemistry of the pulp is altered during regeneration. It is also important to point out that the band appearing at 800 cm^–1^, only for LCP fibers, is most plausibly assigned to lignin’s aromatic C–H bending.^68^ This band can easily be confused with the broad [EMIm]­[DEP] band located at 774 cm^–1^, which is a result of lignin reprecipitation or its reorganization during regeneration.^66^

**Figure 3:**
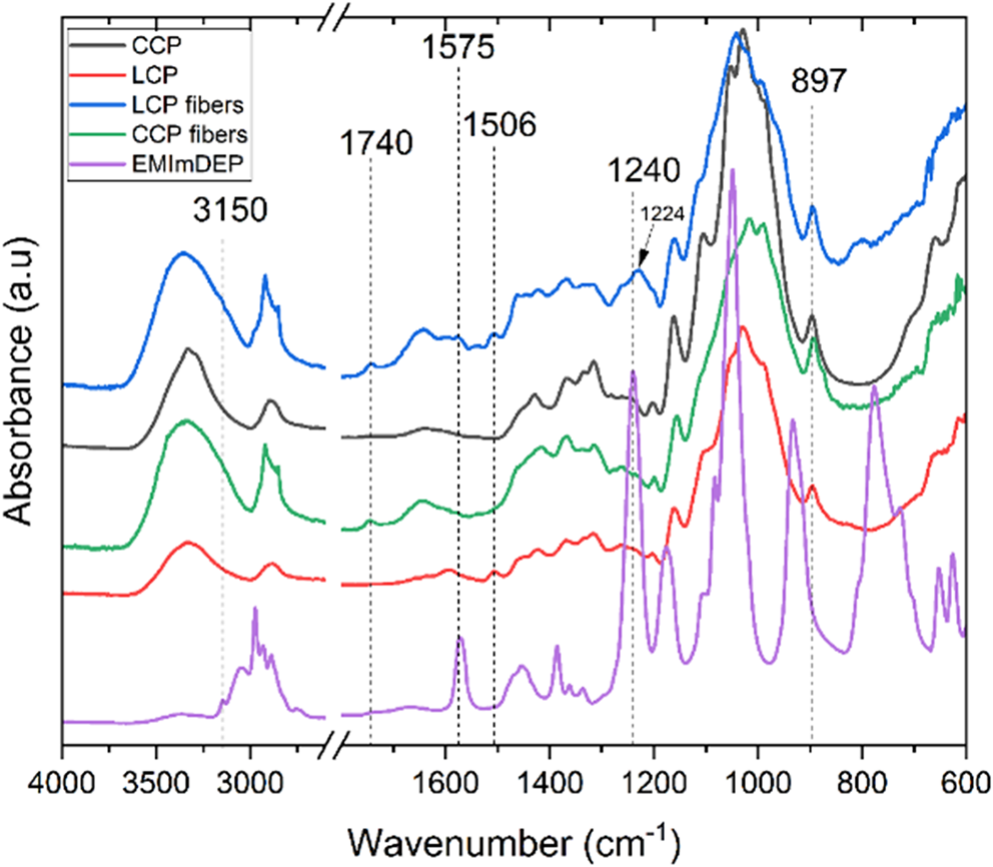
Typical FTIR spectra of cellulose pulps (CCP and LCP), spun fibers (CCP fibers and LCP fibers), and [EMIm]­[DEP].

Wide-angle X-ray diffraction (WAXD) patterns from the fibers are presented in Figure 4. These show both meridional and equatorial intensities characteristic of cellulose and oriented and spun fibers. The fwhm values (Table S1 in Supporting Materials) of the spun fibers calculated from the Lorentz-IV fitted curves show that CCP fibers have values of ∼29°, whereas the LCP fibers return a range of 37–54°. Full-Width Half-Maximum (FWHM)­m is used to evaluate the degree of alignment of cellulose chains, where a lower value (a narrower peak) indicates increased alignment.^9^ The chain alignment of the LCP fibers decreased with increasing LC concentration, with the exception of LCP20. A previous study of similarly spun fibers reported^9,10^ FWHM values in the range 21–29°, which are lower than observed here for the LCP fibers. This difference in FWHM is probably due to the different draw ratios applied and the larger nozzle size used during spinning of the fibers in the present study.

**Figure 4:**
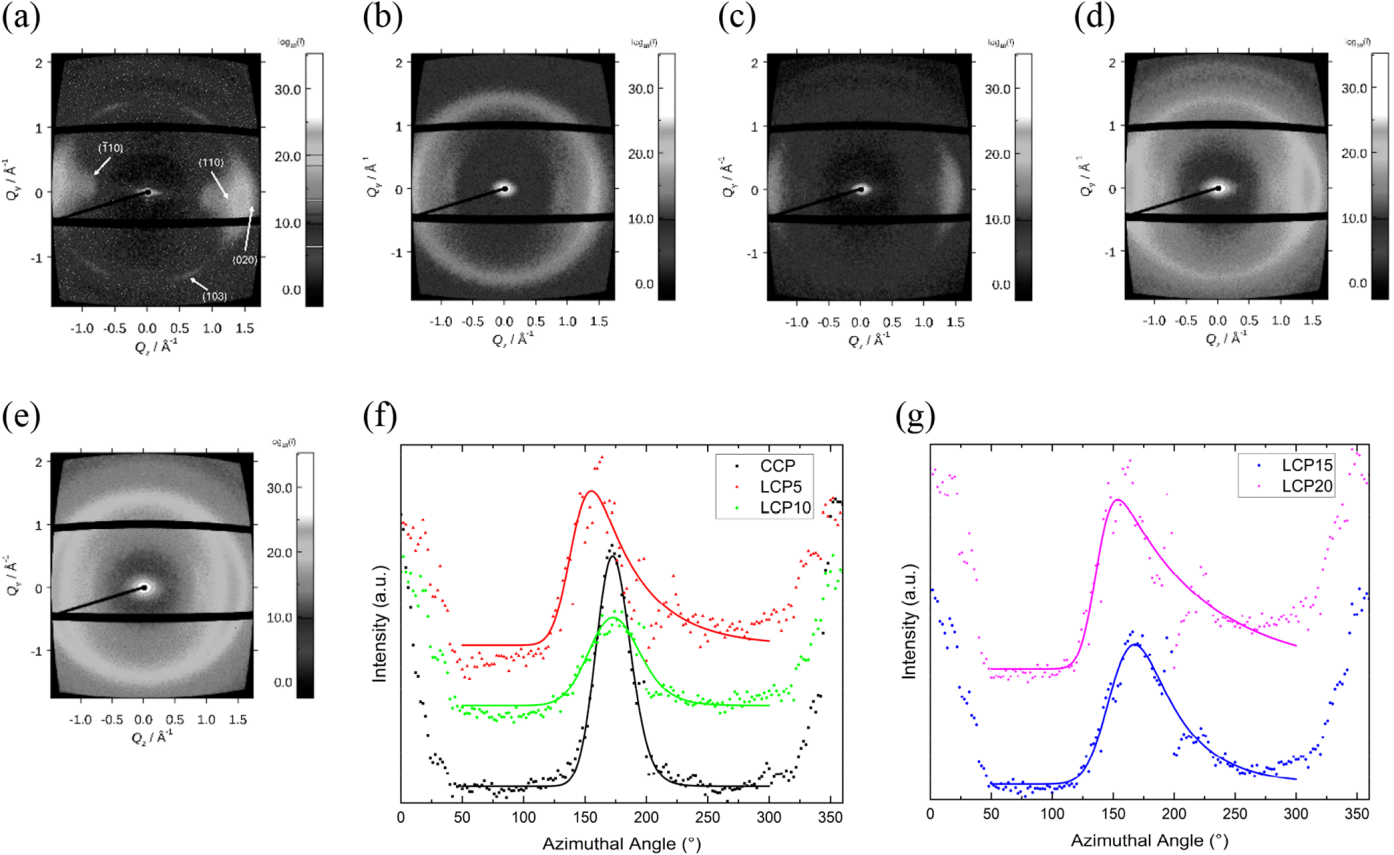
Typical WAXD patterns for (a) CCP, (b) LCP5, (c) LCP10, (d) LCP15, and (e) LCP20 fibers. (f) An azimuthal plot with a fitted equation for CCP, LCP5, and LCP10 fibers and (g) an azimuthal plot with fitted equation for LCP15 and LCP20 fibers. In (f) and (g), the solid lines (red, green, black, purple, and blue) are Lorentz-IV fits to the data. a.u. = arbitrary units.

The extent of the cellulose crystallite orientation in the fibers was characterized using the orientation factor (*f*); these data are presented in Table S1. The equation used to determine *f* from the Azimuthal angle and intensity is as follows^9,10,69^

4 $$f=\frac{\left(3\langle \cos^{2}\theta \rangle -1\right)}{2}$$

where 0 ≤ *f* ≤ 1; a value of *f* = 0 represents alignment perpendicular to the fiber direction, *f* = 0.5 a random orientation, and *f* = 1 completes alignment along the reference axis. Values between 0 and 0.5 are typical for fibers with low crystalline orientation, whereas those in the range 0.6–0.9 have high orientation. Previous studies have reported *f* values in the range of 0.71–0.80 for regenerated cellulose fiber^9,70^ and 0.66 for its composite with multiwalled carbon nanotubes.^70^ Similarly, dry-jet wet-spun cotton waste fiber and birch kraft cellulose fiber were reported^49,50^ to have an orientation parameter in the range of 0.55–0.75, with higher orientation corresponding to the highly drawn fibers (draw ratio >5).

The value of *f* calculated for our fibers indicates that CCP fibers have high crystalline orientation (*f* = 0.80 ± 0.01, Table S1), while the LCP fibers have much lower orientations (*f* = 0.3–0.7, Table S1). This level of orientation is further confirmed by the orientation parameter (<sin^2^ θ>), the value of which indicates a lower orientation of the LCP fibers. A lower value of this parameter is indicative of a higher degree of orientation to the crystalline domains; <sin^2^ θ> = 0.20–0.50 for LCP fibers, and 0.13 ± 0.01 for CCP fibers (Table S1). The relationship between <sin^2^ θ> and <cos^2^ θ> in eq 4 is given in the Supporting Information. The crystallinity index (CrI) of raw miscanthus straw has been reported^71^ to be 65.8%. The spun LCP fibers are expected to have similar values since they are of same chemical composition and previous reports^9,70,72^ have shown that regenerated cellulose fibers have a CrI between 62 and 67%.

### Morphological and Mechanical Properties of the Fibers

Figure 5 presents typical SEM images of the fractured ends of spun fibers postmechanical testing. The CCP spun fibers have a circular cross section (Figure 5a) with a smooth surface along their lengths (Figure 5f). The LCP spun fibers however have a more flattened cross section (Figure 5b) and a much rougher surface (Figure 5g). The “kidney bean” shape of the cross section of the LCP5 fiber section (Figure 5b) is caused by overstretching during processing because of the very low concentration of lignocellulose pulp in the dope. It is worthwhile noting that the low extrusion velocity of 0.62 m s^–1^ was constant for all the spun fibers, resulting in a large diameter to the fibers (120–250 μm). While the CCP fibers have a smooth surface, which is desirable for strength, durability, and aesthetics, the rough surface of the LCP spun fibers presents potential for an enhanced surface area and roughness for contact and adhesion in a composite application.^54^ The smoother surface of the CCP fiber is attributed to the complete dissolution of the commercial cellulose pulp in [EMIm]­[DEP]. The rougher surface of the LCP fibers could be related to a reprecipitation of lignin onto their surface. Complete dissolution was not achieved for the LCP dopes, but remarkably, potentially usable fibers have been produced. The rougher surface of the LCP fibers might be advantageous in an aesthetic sense. What has been produced here is a continuous “natural fiber”, with the texture of common plant filaments. The lack of fiber ends, however, would make these filaments suitable for weaving and other textile processes.

**Figure 5:**
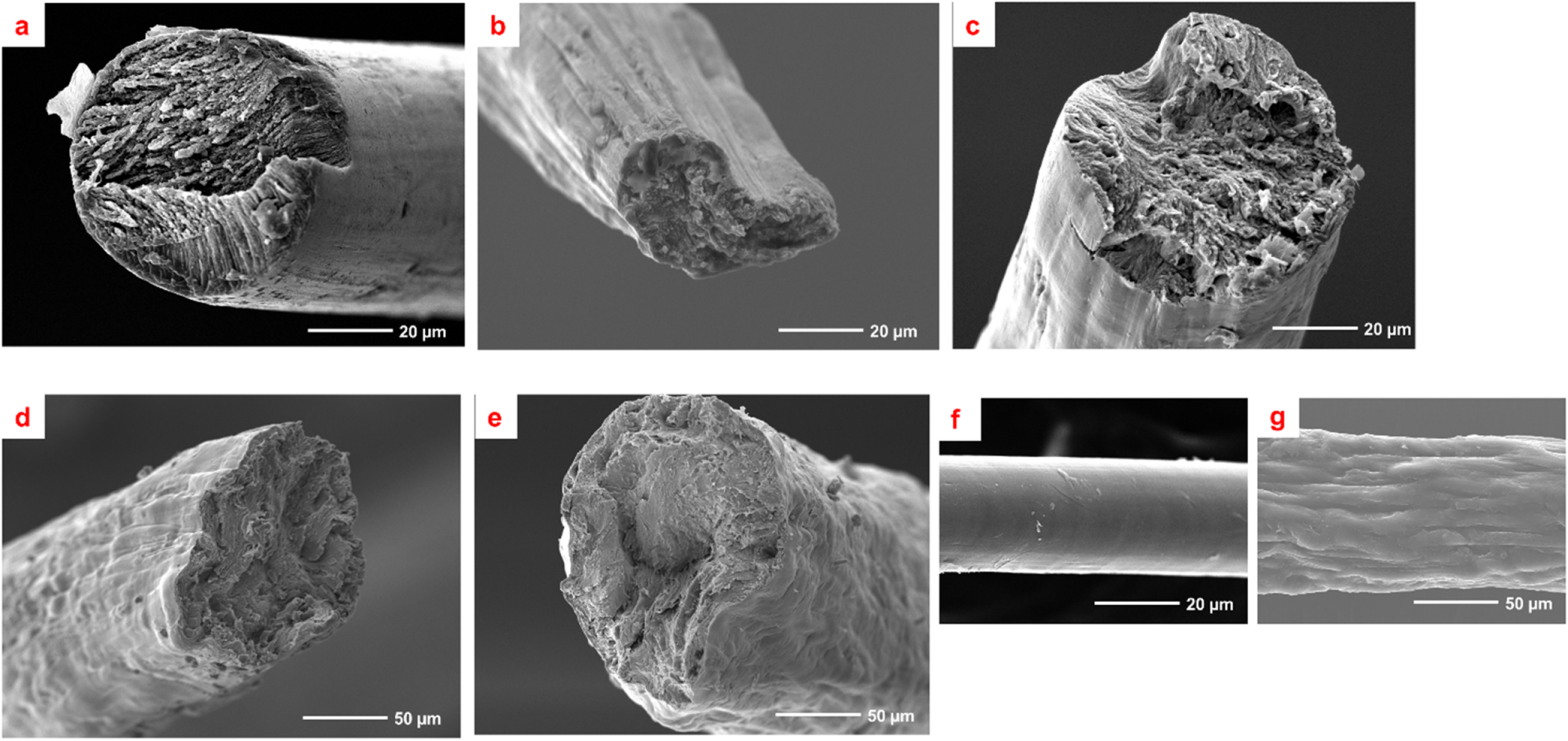
Typical scanning electron microscopy (SEM) images of spun fibers from (a) CCP, (b) LCP5, (c) LCP10, (d) LCP15, and (e) LCP20 precursors; the surface morphologies of (f) a CCP and (g) a LCP fiber.

A summary of the mechanical properties obtained by tensile testing of single filaments of the spun fibers is presented in Figure 6 and Table 2. Figure 6 shows a typical stress–strain curve of the fibers (Figure 6a), the correlation (of different combined fiber gauge lengths) between diameter and tensile strength (Figure 6b), and tensile strength and tensile modulus (Figure 6c) of single filaments. An illustration of the stress–strain curve in Figure 6a shows that the fibers have nonlinear stress–strain behavior, typical of regenerated cellulose fibers. The average elongation at break of the fibers falls within 2.1–2.7% while their yield stress occurs at ∼295 MPa for CCP, ∼128 MPa for LCP10, ∼130 MPa for LCP15, and ∼143 MPa for LCP20 at a yield strain of approximately 1.3%. This yield strain corresponds to the value reported^10,73^ in the literature for regenerated cellulose fibers. Figure 6b demonstrates that the fibers’ tensile strength increases with a decreasing diameter. This is a typically observed relationship, relating to the fact that smaller-diameter fibers will have statistically fewer defects. The group of CCP fibers, having the smallest diameters, possessed the highest strengths (most > 250 MPa). This result is in agreement with the work of Jiang et al.,^74^ who suggested that reduced fiber diameter enhances tensile strength by improved cellulose chain alignment and reduced defects. LCP20 has the largest average diameter of 247 μm and, subsequently, the lowest tensile strength of ∼114 MPa, which also correlates with its much rougher surface (Figure 5e). This rougher surface could result in more stress concentrations, leading to premature failure of the filaments.

**Figure 6:**
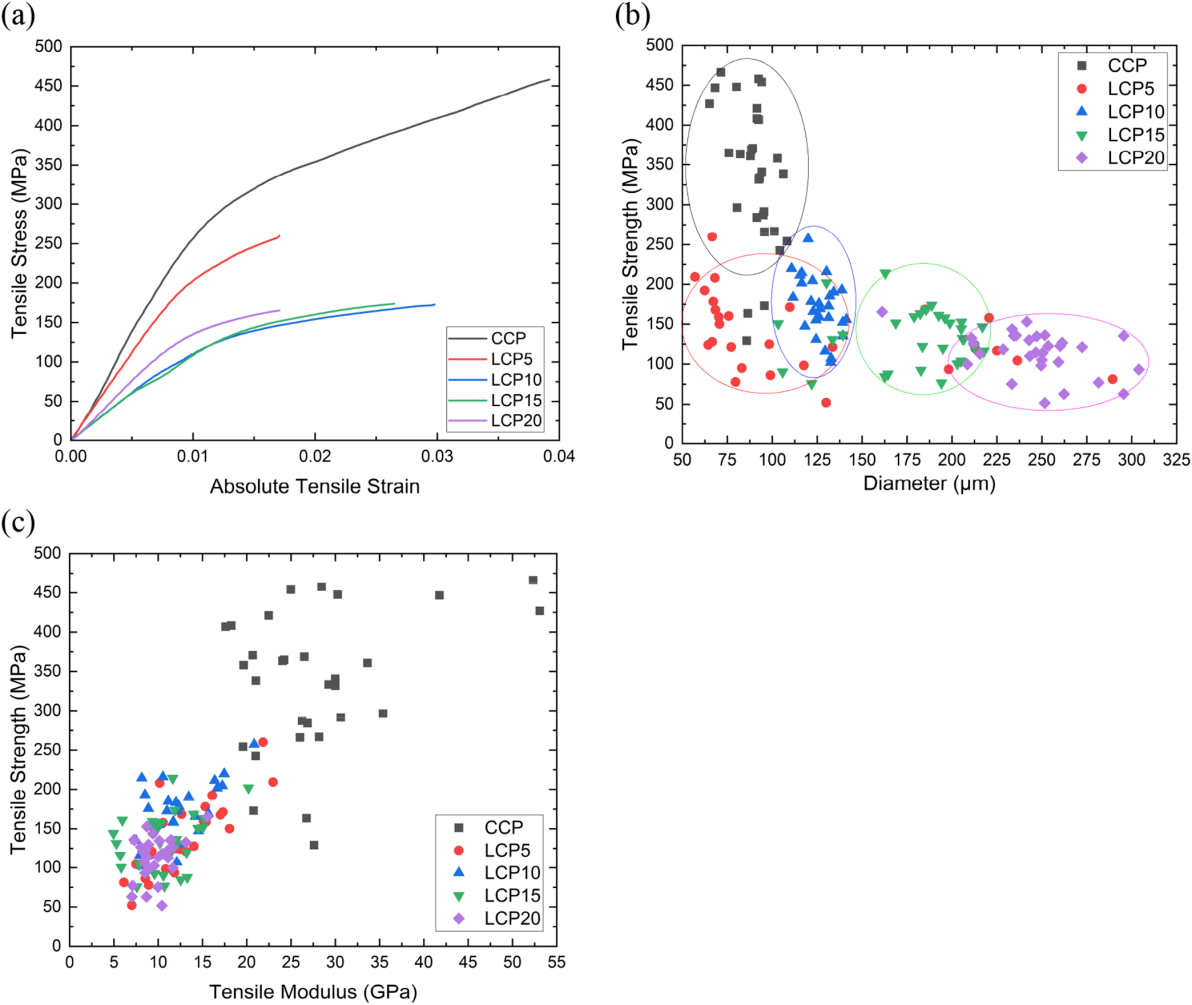
(a) Typical stress–strain curves of the spun fibers. Correlation (of different combined gauge lengths) between (b) the tensile strength of fibers and their diameters and (c) the tensile strength of fibers and their tensile modulus. Different fiber groups are grouped together in (b) (solid lines) as a guide for the eye only.

**Table 2:** Mechanical (Pooled Mean and Pooled Standard Error) and Structural Properties of the Spun Fibers

sample	diameter (μm)	linear density (tex)	tenacity (cN/tex)	tensile strength (MPa)	tensile modulus (GPa)	elongation (%)	FWHM (°)	orientation factor (*f*)	<sin^2^ θ>
CCP fiber	90.0 ± 1.9	8.6 ± 0.3	24.6 ± 1.3	337.4 ± 16.4	27.9 ± 1.6	2.6 ± 0.2	29.1 ± 0.8	0.80 ± 0.01	0.13 ± 0.01
LCP5	119.8 ± 13.1	11.4 ± 1.4	15.3 ± 3.1	138.0 ± 9.2	12.7 ± 0.8	1.8 ± 0.1	37.4 ± 3.5	0.67 ± 0.06	0.22 ± 0.04
LCP10	126.7 ± 1.6	17.1 ± 0.2	12.6 ± 0.5	172.5 ± 6.8	12.2 ± 0.6	2.7 ± 0.2	46.0 ± 3.1	0.52 ± 0.06	0.32 ± 0.04
LCP15	178.3 ± 6.0	44.2 ± 0.6	7.8 ± 0.6	133.9 ± 6.4	10.6 ± 0.6	2.2 ± 0.2	54.2 ± 3.8	0.34 ± 0.09	0.44 ± 0.06
LCP20	246.5 ± 5.3	70.7 ± 3.3	7.6 ± 0.4	114.0 ± 5.0	9.7 ± 0.4	2.1 ± 0.3	42.1 ± 5.1	0.59 ± 0.09	0.27 ± 0.06

Evidently, Table 1 shows that increasing the concentration of the LCP spinning dope resulted in an increased linear density, and a reduced tensile strength and modulus. LCP5 did not follow this trend for tensile strength because of the difficulty in spinning at a very low dope concentration. This also resulted in a wide range of fiber diameters for this sample, from 57 to 289 μm (Table 3). It is notable that a diameter range of 121–159 μm, similar to the LCP fibers produced here, has previously been reported for wet-spun corncob cellulose with an injection needle size of 510 μm.^75^ The internal diameter of the injection nozzle has a significant influence on the physical properties of spun fibers, as it contributes to the orientation and alignment of cellulose chains. It has been reported^76,77^ that smaller-diameter nozzles lead to higher shear rates, which increases the orientation and alignment of cellulose chains, potentially resulting in finer fibers. While the LCP fibers were spun from a nozzle size of 413 μm, previous work^9,10,54,78^ has demonstrated that nozzle diameters in the range of 50–200 μm yield fiber diameters within 5–20 μm. Die swell causes fibers extruded from larger nozzle diameters to have increased diameters due to radial expansion.^79^ This phenomenon impacts the fibers’ dimensions and material properties, resulting in increased probability of defects and a coarser microstructure.^80,81^ It is worthwhile noting that given the incomplete dissolution of the biomass occurring for the LCP fibers, a smaller nozzle size might result in blockages occurring, ultimately preventing spinning from taking place.

**Table 3:** Statistics on the Measurements of the Diameters of the Spun Fibers^a^

name	minimum	maximum	standard deviation	SE of mean	coefficient of variation	normality test *p*-value
CCP fiber	65.1	108.2	10.5	1.9	0.1	0.2
LCP5	57.0	289.4	67.8	13.1	0.6	(0.00018)
LCP10	110.8	141.2	8.4	1.6	0.1	0.6
LCP15	103.4	217.8	32.6	6.0	0.2	(0.0033)
LCP20	161.2	304	28.8	5.3	0.1	0.2

a All values are in microns. Values in brackets when <0.05. The calculated ANOVA *p*-value of <0.0001 indicates that the diameters for all the fibers are significantly different from each other. LCP5 and LCP15 with normality test *p*-value < 0.05 show that their diameters are not normally distributed like other fibers.

Figure 6c illustrates the relationship between the tensile strength and the modulus of the fibers, which gives a map of the potential properties. The LCP fibers exhibited lower strengths and moduli compared to the CCP fibers. A more complete dissolution of the CCP in the IL could contribute to an enhanced chain alignment, resulting in an increased strength and modulus. Generally, the moduli and strengths of the LCP fibers fall within the ranges 5–15 GPa and 50–250 MPa, respectively. Low tensile strengths of 73 to 130 MPa have previously been reported^82^ for spun microcrystalline cellulose. Regenerated cellulose fibers obtained from wet spinning of dissolved corncob cellulose in a CO_2_ switchable solvent also had low modulus and strengths of 12.5–89.9 cN/dtex (∼1–9 GPa) and 0.21 to 1.05 cN/dtex (∼23–116 MPa) despite complete dissolution of the cellulose.^75^ It is notable that the moduli and strengths of the LCP fibers are similar to some commercial textile grades,^83^ including Lyocell (∼15 GPa, ∼500 MPa) and Enka Viscose (∼9 GPa, ∼220 MPa). Fibers spun from steam-exploded aspen wood, directly dissolved in the *N*-methyl-morpholine-N-oxide (NMMO) solvent^84,85^ (used for Lyocell production), yielded similar mechanical properties to those achieved here (9–14 GPa, 200–300 MPa), although with much smaller-diameter filaments (∼20 μm). We recognize that these properties are not the highest among regenerated cellulose fibers, but our aim here was to retain as much of the lignocellulosic material as possible in the spinning process, without the need for fractionation. If purer cellulose materials are used, one can make filaments of much higher strength and stiffness, but here we achieve a compromise in performance, while retaining carbon.

### Thermal Stability of the Spun Fibers

The thermal degradation of the dry-jet wet-spun fibers and the precursor pulps was measured under a nitrogen atmosphere, the results of which are shown in Figure 7. Figure 7 combines thermogravimetric (TGA) curves, which show the mass change of the spun fibers over temperature, and derivative thermogravimetric (DTG) curves, showing the rate of mass change with temperature. The TGA curve (Figure 7a) shows 3 stages for the thermal degradation for all the spun fibers, which is in agreement with the thermal decomposition of many regenerated cellulose fibers^54,70,86–88^ and polyethylene-based carbon fiber precursors.^88,89^ The spun fibers showed a more rapid loss of weight during the drying stage than the pulp precursors. This loss continued until more than 5% (*T*_5%_) of their weight was lost between 118 and 156 °C. The pulps were however able to withstand temperatures of ∼250 and ∼262 °C, for LCP pulp and CCP, respectively, before the same weight loss occurred (Table S2). This initial 5% loss in weight is normally attributed^33,90,91^ to the loss of surface moisture and low-molecular-weight compounds. The *T*_5%_ of the spun fibers is lower than that of the pulps, which is believed to have resulted from the air-drying of the spun fibers. However, during the pyrolysis stage, the maximum decomposition peak temperature (*T*_max_) of the spun fibers falls within the range 329–337 °C. This is close to the *T*_max_ of the pulps: ∼317 °C for LCP pulp and ∼353 °C for the CCP pulp. This confirms that the spun fibers did not decompose at *T*_5%_; rather, there was a removal of the remaining absorbed water from the washing step. It is important to mention that rapidly drying the spun fibers, or ensuring complete moisture loss, could denature them, causing embrittlement.

**Figure 7:**
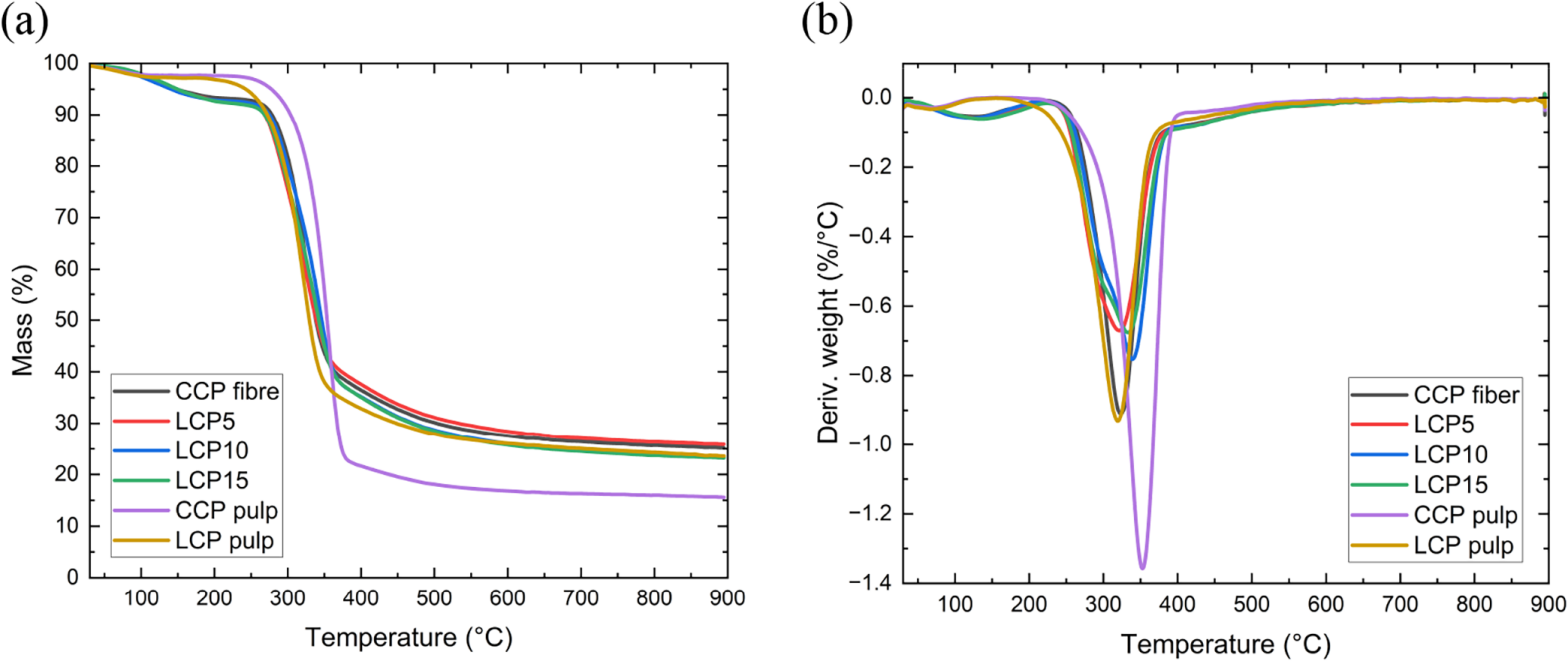
(a) TG and (b) DTG curves of the dry-jet wet-spin fibers and their precursor pulps.

The TGA thermogram (Figure 7b) shows that about ∼13% residue of the CCP pulp remained after the carbonization stage at 800 °C. Surprisingly, this is 46% lower than the residue remaining from LCP20, which has the lowest residue among the spun fibers. This may have arisen from the presence of lignin in the LCP pulp and fibers and an increased surface area of the pure cellulose favoring depolymerization into volatiles rather than char. Regenerated cellulose fibers are known to have a lower surface area compared to raw pulp because of their more compact and ordered structure of the crystalline regions. This reduces the available surface for thermal interactions, leading to enhanced thermal stability and slower thermal degradation rates, as there are fewer sites for thermal reactions to occur.^92,93^

The spun fibers and LCP pulp have residues within the same range, 19–23% (Table S2). Understandably, the LCP pulps have more residue than the CCP pulp, which can be attributed to the purity of the latter. The thermal decomposition of the LCPs must have allowed for more complete conversion of the cellulose into char rather than volatile products, especially when influenced by the presence of hemicellulose and lignin.^93^ Notably, the quantity of residues of the LCP spun fibers at 800 °C was within a narrow range of 19–22%. Since this range is within the margin of error, it shows that increasing LCP concentrations may not lead to a higher carbon yield during carbon fiber production. Dumanlı et al.^94^ explained that despite the theoretical carbon yield of the carbonization process for cellulose being 44.4%, the actual yield is typically only between 10 and 30%. The spun fibers produced in this work fall within the mid limit of this range. The reason for this low carbon yield has been attributed^95^ to depolymerization of the macromolecular chains, and oxygen taking away carbon as carbon monoxide (CO) and carbon dioxide (CO_2_). Amelioration of this loss can be achieved by reducing the heating rate of the carbonization process^94^ to about a few °C/h^–1^, impregnation of the fibers with phosphate salts,^96^ sulfuric acid treatment^97^ of the fibers, or dehydrating the fibers with methanol^98^ before carbonization. Future work will address the carbonization of these LCP fibers and their use in composite materials.

## Conclusions

Dry-jet wet spinning of partially treated lignocellulose pulp and the effect of undissolved dope particles on subsequent fiber properties have been demonstrated. [EMIm]­[DEP] has been shown to be a good and stable solvent for the dissolution of pure cellulose but does not allow complete dissolution of all lignocellulose components in one pot, under the investigated conditions. A complete dissolution of these components has however been shown not to be necessary if mid-range mechanical properties are desirable. This approach could be used for applications where continuous fibers are required at low cost and to also retain carbon in their processing. The incomplete dissolution of the lignocellulose pulps posed a challenge of having a very viscous spinning dope at ambient temperature as the concentration of the pulp in the IL increases. However, the shear thinning behavior of the dopes and a temperature of 80 °C ensured the dopes were spun into continuous filaments. The LCP fibers exhibited a rough surface with noncircular cross sections and larger diameters than the CCP fibers, ranging from 120 to 250 μm. Despite the low draw ratios and larger nozzle size needed for spinning, the LCP fibers achieved a tensile strength of about 173 MPa, and an appreciable modulus of about 9–15 GPa, comparable to that of some commercial grades of fibers. There is potential for the improvement of these properties, perhaps with a reduced fiber diameter and a subsequent increase in orientation and chain alignment. The thermal stabilities and char residues for the LCP fibers were promising, showing a higher end of a yield among cellulose carbon fiber precursors. Therefore, this study demonstrates that lignocellulose biomass may not require rigorous pretreatment to achieve significant properties for technical textiles and carbon fiber applications.

## Supplementary Material



## Notes

Typical optical microscopy images of the lignocellulose spinning dopes LCP5, LCP10, LCP15, and LCP20; relationship between ⟨sin^2^ θ⟩ and ⟨cos^2^ θ⟩ (Figure S1); mechanical properties of the spun fibers showing the effect of gauge length at constant strain rate (0.125 min^–1^) on their tenacity, strain at break, tensile strength, and tensile modulus; statistical difference for each fiber property within the 3-gauge length for each fiber type (Figure S2); TGA and DTG analyses of the spun fibers and their precursor pulps (Table S1); statistics of the linear density measurements for the spun fibers, with *p*-values showing that only LCP5 does not have a normal distribution (Table S2) (PDF)
