# Supplementary material for: Looking Beyond Pure Cellulose to Lignocellulose for Regenerated Continuous Spun Filaments
Source: ACS Omega. 2025 Dec 13;10(51):63583–96. doi: 10.1021/acsomega.5c10782 (PMC12756835; doi:10.1021/acsomega.5c10782)
Supplement: Supplementary file 1 [file ao5c10782_si_001.pdf]

SUPPLEMENTARY INFORMATION

Looking Beyond Pure Cellulose to Lignocellulose for Regenerated Continuous Spun Filaments

Chinomso M. Ewulonu[1], *Stefania Akromah*[1], *Koon-Yang Lee*[2], *Annela M. Seddon*[3], *Cariny Polesca*[4], *Jason P. Hallett*[4], *Stephen J. Eichhorn*[1,5*]

1. Bristol Composite Institute, School of Civil, Aerospace, and Design Engineering, University of Bristol, Bristol, BS8 1TR, United Kingdom.

2. Department of Aeronautics, Imperial College London, South Kensington campus, London SW7 2AZ, United Kingdom.

3. H.H. Wills Physics Laboratory, School of Physics, University of Bristol, Bristol, BS8 1TL.

4. Department of Chemical Engineering, Imperial College London, South Kensington Campus, London, SW7 2AZ, United Kingdom.

5. School of Chemistry, Faculty of Science and Engineering, Cantock's Close, University of Bristol, Bristol, BS8 1TS, UK.

*Corresponding Author: s.j.eichhorn@bristol.ac.uk

Physical Properties of the Spinning Dope

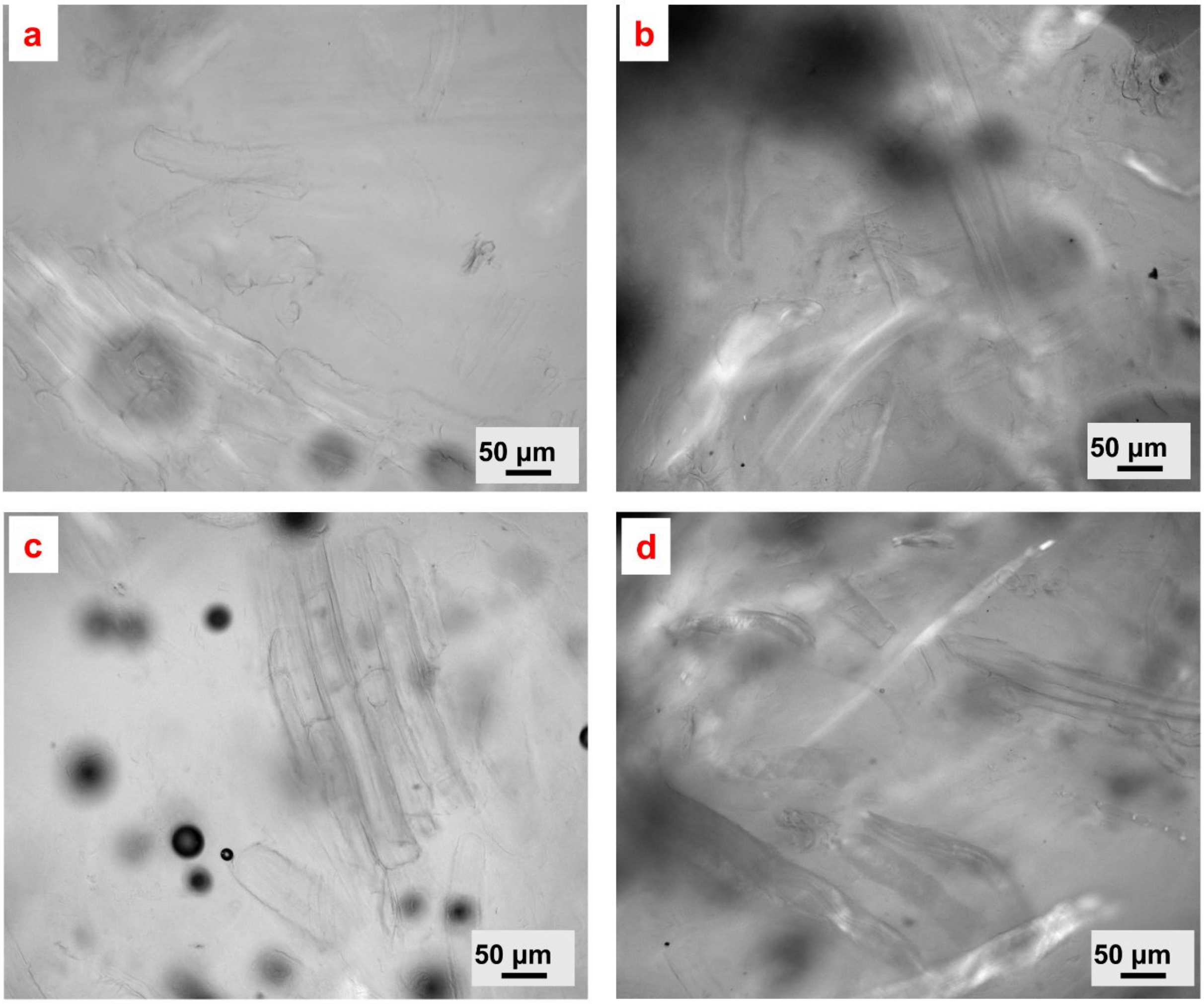

Figure S1: Typical optical microscopy images of the lignocellulose spinning dopes (a) LCP5, (b) LCP10, (c) LCP15, and (d) LCP20.

Mechanical and structural properties of the spun fibers

The relationship between $\langle \sin^2\theta \rangle$ and $\langle \cos^2\theta \rangle$ in equation 4 is given by the equations

$$\langle \sin^2 \theta \rangle = 1 - \langle \cos^2 \theta \rangle \qquad \text{(S1)}$$

$$\langle \sin^2 \theta \rangle = \frac{\sum I(\theta) \langle \sin^2 \theta \rangle \Delta\theta}{\sum I(\theta)} \qquad \text{(S2)}$$

where θ is the reference angle (the fiber axis or peak maximum) and I is the normalized intensity distribution from the azimuthal scan in WAXD. The azimuthal profile can be fitted to a Lorentz-IV distribution to calculate $\langle \sin^2 \theta \rangle$ as follows[1]:

$$\langle sin^2\theta \rangle = \frac{0.4}{\rho^2} \qquad \text{(S3)}$$

$$\rho^2 = \frac{0.1892}{sin^2\theta_h} \qquad \text{(S4)}$$

where θ_h is the half-width at half-height obtained from the Lorentz-IV fitting.

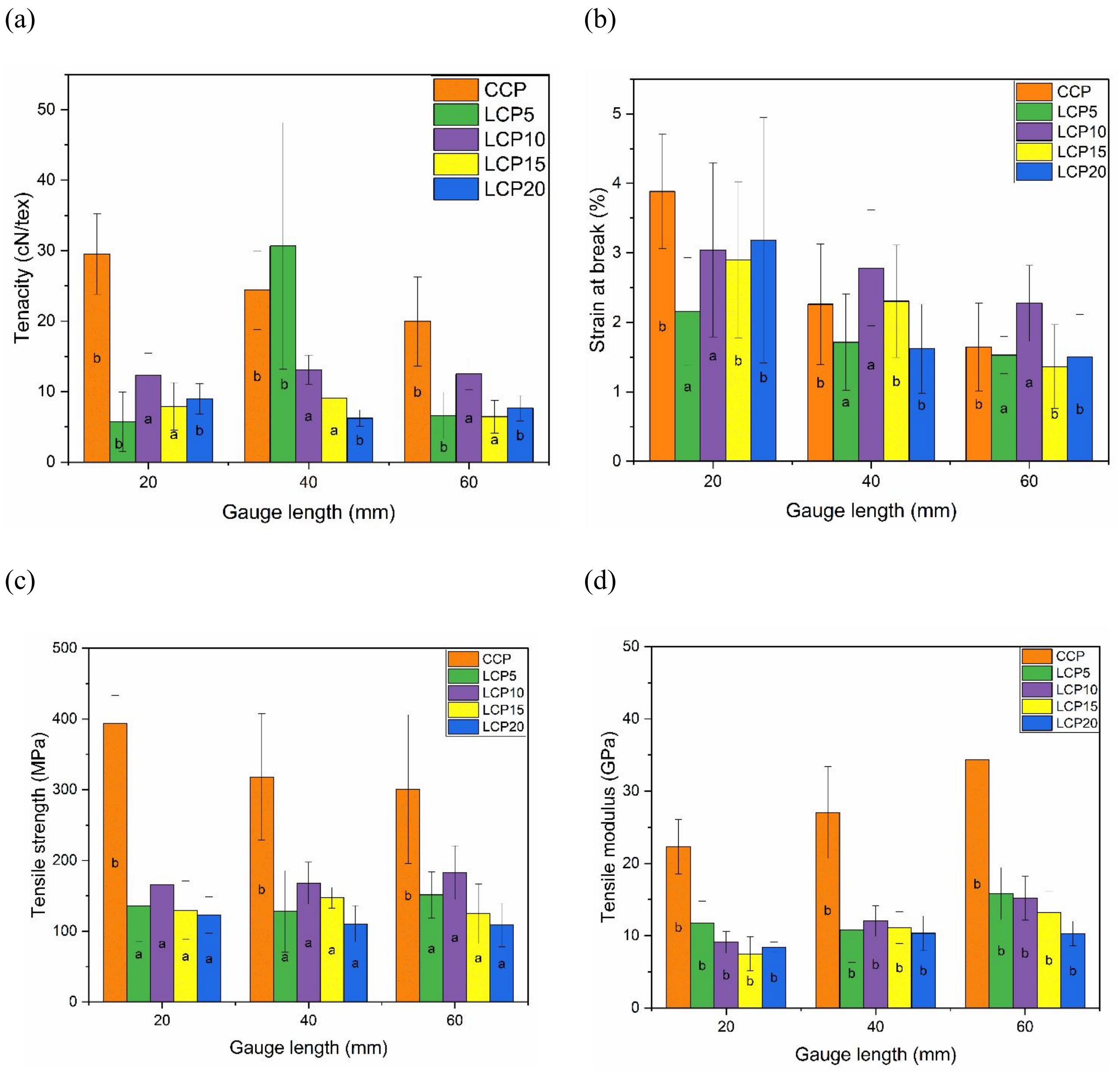

Figure S2: Mechanical properties of the spun fibers showing the effect of gauge length at constant strain rate (0.125 min^{-1}) on their (a) tenacity, (b) strain at break, (c) tensile strength, and (d) tensile modulus. Statistical difference for each fiber property within the 3-gauge length for each fiber type is represented as a – significantly different (ANOVA p-value <0.05) and b – not significantly different (ANOVA p-value >0.05).

Table S1: Thermal (TGA and DTG) analyses of the spun fibers and their precursor pulps.

Sample	TGA analysis		DTG Analysis	
	$T_{5\%}$ (˚C)	Residue at 800 ˚C (%)	T_{max} (˚C)	Residue at T_{max} (%)
CCP fiber	117.9 ± 30.0	20.1 ± 4.9	330.8 ± 6.9	58.2 ± 4.3
LCP5	130.9 ± 17.1	21.4 ± 4.8	329.3 ± 7.2	57.6 ± 3.7
LCP10	125.3 ± 14.6	22.0 ± 1.8	342.0 ± 3.2	53.5 ± 2.4
LCP15	148.7 ± 3.7	22.0 ± 1.5	337.4 ± 3.6	54.0 ± 2.2
LCP20	156.4 ± 22.3	19.2 ± 2.0	337.6 ± 1.7	51.7 ± 0.9
CCP pulp	262.3 ± 14.4	13.2 ± 2.4	353.9 ± 1.2	49.6 ± 3.1
LCP pulp	250.1 ± 6.4	23.0 ± 1.4	317.3 ± 2.3	58.3 ± 1.5

Table S2: Statistics of the linear density measurements for the spun fibers. The p-values show that only LCP5 does not have a normal distribution.

Name	N total	Minimum (tex)	Mean (tex)	Maximum (tex)	Standard Deviation	Coefficient of Variation	Normality test p-value
CCP	20	4.6	8.62	11.8	1.83	0.21	0.74
LCP5	20	4.2	11.36	33.2	8.16	0.72	0.00028
LCP10	20	14.4	17.06	19.6	1.34	0.08	0.98
LCP15	20	36.6	44.23	50	3.21	0.07	0.83
LCP20	20	42.8	70.73	113.2	18.54	0.26	0.13

References

(1) Northolt, M. G. Tensile Deformation of Poly(p-Phenylene Terephthalamide) Fibres, an Experimental and Theoretical Analysis. *Polymer (Guildf)* 1980, *21* (10), 1199–1204. https://doi.org/10.1016/0032-3861(80)90088-9.
